# Implementation of fuzzy logic control algorithm for temperature control in robusta rotary dryer coffee bean dryer

**DOI:** 10.1016/j.mex.2024.102580

**Published:** 2024-01-26

**Authors:** Nihayatun Nafisah, Ika Noer Syamsiana, Ratna Ika Putri, Wijaya Kusuma, Arwin Datumaya Wahyudi Sumari

**Affiliations:** a Department of Electrical Engineering, State Polytechnic of Malang, Malang, 65141, Indonesia; b Faculty of Industrial Technology, Adisutjipto Institute of Aerospace Technology, Yogyakarta 55198, Indonesia

**Keywords:** This article uses the fuzzy logic control method with mamdani interference to regulate the temperature in a rotary dryer, Control engineering, Coffee quality, Rate of drying, Moisture content

## Abstract

Background: Indonesia is one of the coffee producers ranked third in the world in the supply of coffee beans. To maintain competitiveness international market, it is necessary to maintain and improve the quality of coffee beans.

Objective: One crucial aspect of maintaining the quality of coffee beans is maintaining the moisture content of green coffee beans. One of the water content settings is using the drying method. While traditional drying methods often experience weather and long-time constraints.

Results: This study designed an innovative coffee bean dryer based on fuzzy logic to overcome the problem. This system uses temperature control with Mamdani's fuzzy logic control interference algorithm, input and delta errors, and output percentage valve opening. This method achieved a moisture content following SNI standards of 12% and a 0.00015% / s drying rate for each coffee bean mass increased by 1kg. This method is also more efficient and stable in maintaining the temperature at a value of 50°C.

Methods: The drying equipment also estimates the drying time by considering variations in the mass of coffee beans. This dryer can provide an effective solution to maintain optimal coffee bean quality.

Conclusion: The second semi-wash method of drying coffee beans using a fuzzy logic-based coffee bean drier has proven successful for drying coffee beans to a moisture content of 12% in a period of 90 min to 195.65 min with a drying capacity of 1 kilogram to 10kg at 50°C.

• The coffee beans utilized in the studies are robusta coffee beans from plantations on Mount Kawi's slopes in East Java, Indonesia.
• The trial sample was 1 kilogram of green coffee beans removed from the horn skin.
• According to SNI standards, the drying performed is the second in the postharvest semi-wash procedure to achieve a moisture content of 12%.

## Graphical abstract

**Figure:**
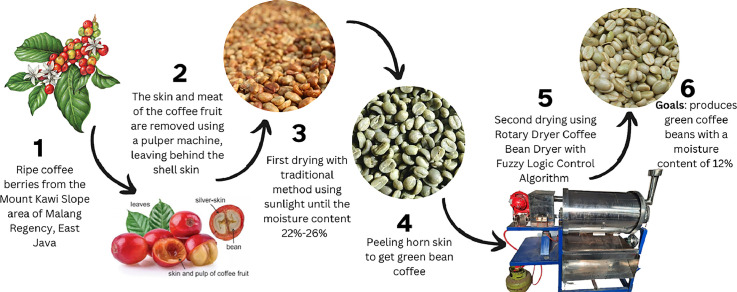

Specifications table

**Table:** 

Subject area:	Engineering
More specific subject area:	Control System Engineering
Name of your method:	This article uses the fuzzy logic control method with mamdani interference to regulate the temperature in a rotary dryer
Name and reference of original method:	Sutrisno, D. Ariwibowo, M. E. Yulianto, and R. Sitawati, “Characteristic of Vertical Mixed Flow Dryer in Coffee Bean Drying Process,” IOP Conf. Ser. Mater. Sci. Eng., vol. 771, no. 1, p. 012,070, Mar. 2020, doi:10.1088/1757-899X/771/1/012,070
Resource availability:	Ms. Office 2021, Matlab 2021, Fritzing, Arduino Ide, Sketchup, Ms.Visio

**Methods details**

## Introduction

According to the International Coffee Organization (ICO), world demand for coffee beans increased by 3% in 2021 [1,^2^,3]. Indonesia is the world's third-largest producer of coffee beans, following India and Brazil [4,^5^,6]. The demand and supply of coffee beans in Indonesia not only reached the domestic market but have expanded beyond consumers worldwide [7,^8^,^9^,10]. Trading in low-grade coffee has been prohibited since October 1st, 2002 according to resolution 407 by the ICO [11]. One indicator of high-quality coffee beans is having a moisture content lower than 12.5% and a pleasant odor without any musty smell [12,^13^,14]. Based on Indonesian National Standart (SNI) 01–2907–2008, post-harvest processing is necessary after harvesting coffee beans. Post-harvest processing method affects the quality of coffee beans [15,16].

Drying is a post-harvest technique that removes moisture from freshly harvested crops or produces [17,18]. This process helps to extend the shelf life of the crops and prevents the growth of fungi and bacteria that can cause spoilage [19,^20^,17]. There are two methods for drying coffee beans: traditional and mechanical. [21]. The traditional method involves drying in direct sunlight [22,^23^,^24^,25]. However, this method has several drawbacks, including a longer drying time, reliance on the weather, and the increased likelihood of fungi and bacteria contamination of coffee beans [26,^24^,27].

Drying wet coffee beans in direct sunlight takes 5–7 days, reducing moisture content from 45 to 50% to 18–20%. After removing the horn skin, it is dried for an additional two days to reach a moisture content of 11–12.5%. In Indonesia, coffee beans are often dried suboptimally due to high rainfall, erratic weather, and humidity in highland areas [28,^29^,30]. Traditional drying of coffee beans in direct sunshine with or without a base in the open air encourages the growth of 10 to 13 varieties of fungus [31,32].

Due to some of the negative consequences of drying coffee beans in conventional ways, it is needed to fix these problems by using a drying machine as an option [33]. Mechanical drying of coffee beans with air heated to 50°C achieves excellent coffee with better chemical and physical qualities than direct solar drying [34,^35^,36]. Furthermore, mechanical drying with machines contains more sucrose and less fatty acids than direct drying with sunlight, making it healthier than conventional drying [34].

Much research has been completed to enhance the drying processes for coffee beans. Previous research has concentrated on temperature regulation. One emphasizes the effectiveness of applying a controlled drying oven to dry coffee beans. However, even though the temperature has been controlled to a certain set point, drying in this oven has the drawback of uneven drying of the coffee beans [37]. Another study uses a YSD-UNIB18 hybrid drier to dry robusta coffee beans using a thin-layer drying kinetics fitting model. In this research, an appliance similar to an oven with thin shelves organized in layers was utilized to equally spread the dryness of coffee beans over each tier. The fuel applied is firewood, which generates pollutant emissions that damage the environment. Furthermore, energy resources are limited, and not all locations have an abundance of firewood. Firewood fuel is inefficient and has a significant risk of contaminating coffee beans [38].

The following study will use the vertical mixed flow technique to dry coffee beans. However, no control mechanism-controlled temperature and time in this study. It is not specified which controls are utilized. Therefore, it is likely to be semi-manual [39]. In addition, another drying method uses a semi-spherical solar dryer of arabica coffee beans. The study utilized a hybrid heat source for drying coffee beans. Solar energy was used during the day, while biomass was used at night. However, the tool lacks a temperature controller to regulate the temperature based on set points. Moreover, drying the coffee beans takes 16–17 h to reach 12% moisture content [40].

Based on existing research, it is essential to develop a coffee bean dryer that can dry coffee beans effectively and control temperature and duration to create coffee beans with standard moisture content. Our primary target is to create a horizontal rotary dryer by controlling the temperature and eventually producing coffee beans with moisture content less than 12.5% according to SNI requirements. Although there have been many studies on drying coffee beans through various methods, this study tries to offer another and unique perspective by using fuzzy logic control as temperature control, rotary dryer as drying media, gas stove as a source of dryer heat energy, and time estimation based on the mass of dried coffee beans so that the final result of dry coffee beans is less than 12.5% according to standards. When implementing the conventional technique, the second drying using the semi-wash method takes two to three days; thus, this appliance significantly helps minimize drying time and results in coffee bean moisture content that meets the criteria.

## Material and method

This study took place at the State Polytechnic of Malang's AK Building Control Laboratory and the Power Laboratory of the Department of Applied Master of Electrical Engineering. The research was conducted between September 2022 and November 2023. The object of research is Gunung Kawi Robusta coffee, which is being studied using an ESP32 microcontroller and an automated dryer with temperature controls.

### Material

Robusta coffee beans (*coffea canephora*) are utilized, and they are picked directly from coffee bean cultivators on the Mount Kawi Slope area of Malang Regency, East Java. The robusta coffee beans applied as research samples are green coffee beans that have been semi-washed method after harvest. After the horn skin is peeled, green coffee beans exist, and the drying used in this study is the second drying. The number of green coffee beans used as experimental samples had a mass of 1kg each with an initial moisture content of 23.5% and three-time intervals of 30 min, 60 min, and 90 min.

### Semi wash method

In this method, the skin and meat of the coffee fruit are removed using a pulper machine, leaving behind the shell skin [41]. After that, the peeled coffee beans will be placed in a bag for almost one night to remove any remaining mucus from the coffee [42,43]. Then, the coffee beans are dried by two drying processes [44]. The first step in drying coffee beans with shells is through direct sunlight [45]. Coffee that is still in a semi-dry state will be peeled off the horn skin with a machine called a huller, and the process itself is called hulling [46]. After the first drying, the moisture content of coffee beans is 22% - 26% [47].

### Rotary dryer

**Fig. 1:**
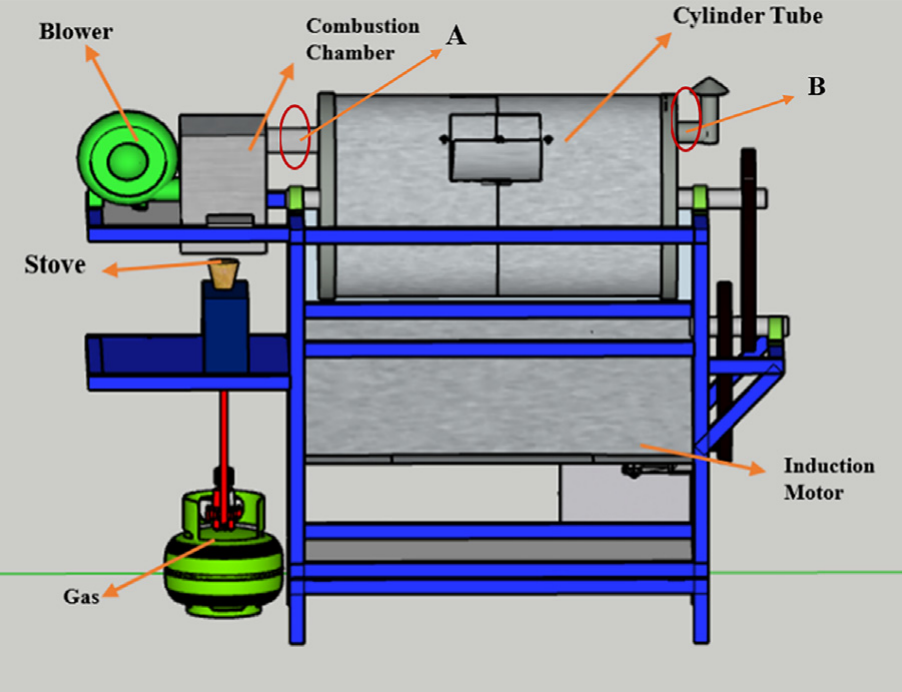
3D Coffee bean dryer design.

A rotary dryer, often known as a drum dryer, is a dryer that rotates continuously [48,49]. The dryer in this research is heated by hot air shouted by a blower from the heating burner [50,51]. Direct contact with hot air moving countercurrent with a stream of solid material is used to heat coffee bean [52,53]. Drying using the rotary dryer method has the advantage of drying coffee beans evenly and thoroughly to the inner layer of coffee beans [54,55]. The specifications of the drying tube in this study use stainless steel material with a thickness of 2mm, a rotary diameter of 40cm, and a length of 67cm. In addition, the alphabet A in Fig. 1 is the place to place the DHT22 sensor for the inlet temperature and the alphabet B is the place to place the DHT22 sensor for otlet temperature. The design of the drying tube can be seen in the Fig. 1.

### Dry matter content

According to SNI 01–2907–2008, coffee beans are considered high-quality if they have a moisture content of 12.5% or less [12]. To measure the water activity of coffee beans, a grain meter with plate number MD7822 is used. It has a moisture content measurement range of 2% to 30%, and readings are taken at a temperature of 25±1°C. In addition, to validate the accuracy of the MD7822 series grain meter, the gravimetric method is used which is one of the analytical methods to determine the quantity value of a substance by measuring the weight of the substance in a pure state after going through the separation process [56,^57^,^58^,56]. In this case, the type of gravimetric analysis used is evaporation to obtain the difference in the mass of coffee beans before being fertilized and after evaporating until completely dry using the oven [59]. The equation to obtain the value of moisture content using the gravimetric method is contained in the Eq. (1):

(1) $$\%Moisture\;Content=\frac{sample\;weight\;change}{original\;sample}\times \;100\%$$

### Block diagram of tool work system

**Fig. 2:**
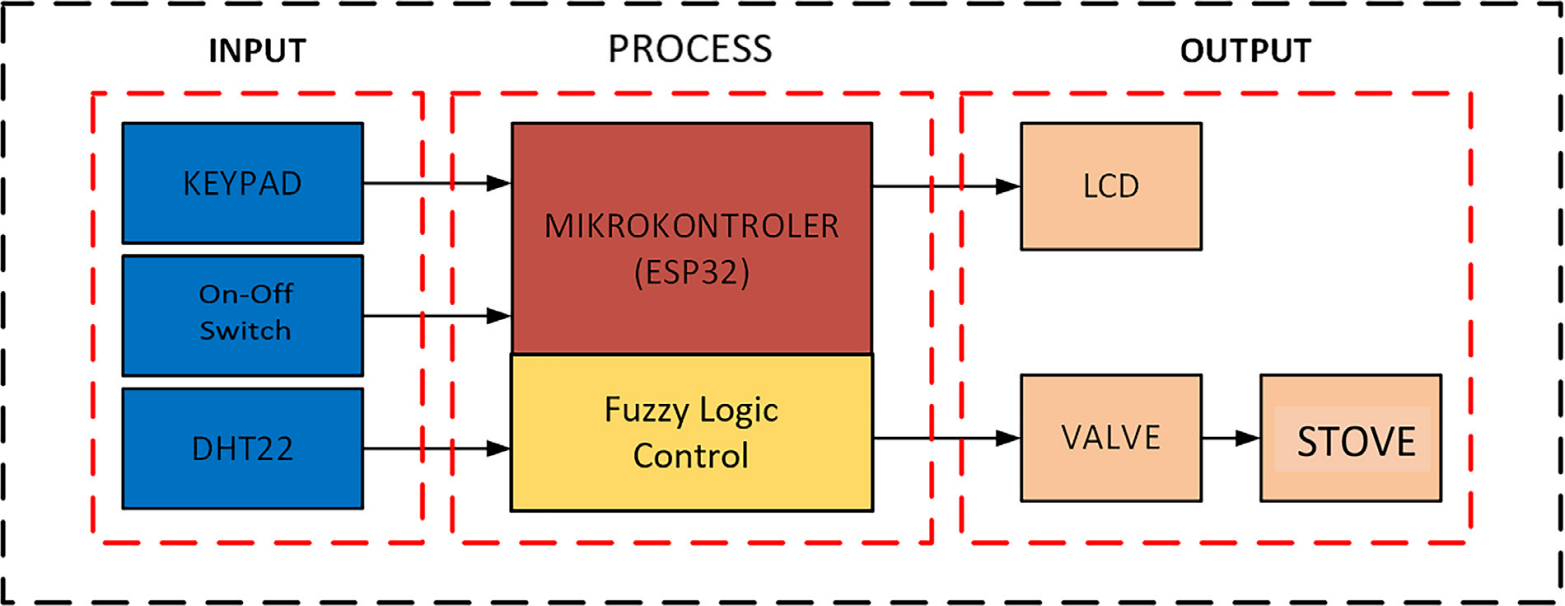
System block diagram.

The design and assembly of tools linked into a single work system is made up of three components: input, controller (process), and output. Input is a component of the system that works to provide commands to the controller in the form of voltage to the microcontroller. The process is a system component that processes and executes commands from input with a specified program. In comparison, the output is a component of a system that serves to accept orders from the process and carry them out in line with the functions of the designated equipment. Fig. 2 depicts a block schematic of the system architecture for the drying of coffee beans.

**Fig. 3:**
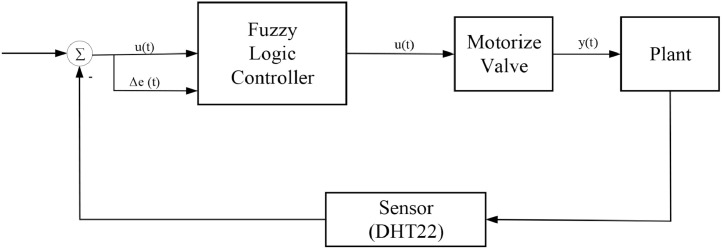
Control block diagram.

The drying of coffee beans is controlled by a control system that utilizes fuzzy logic control. The first input is the error value calculated by subtracting the set point value (initial temperature) from the current value (set point temperature). The temperature sensor (DHT22) is used to determine real-time the temperature value. The delta error value (error - last error) is the second input. The fuzzy logic controller technique produces a PWM (pulse width modulation) value that adjusts the valve opening. The temperature sensor (DHT22) value will be utilized as feedback to determine the error value for each iteration that has been conducted (Fig. 3).

**Fig. 4:**
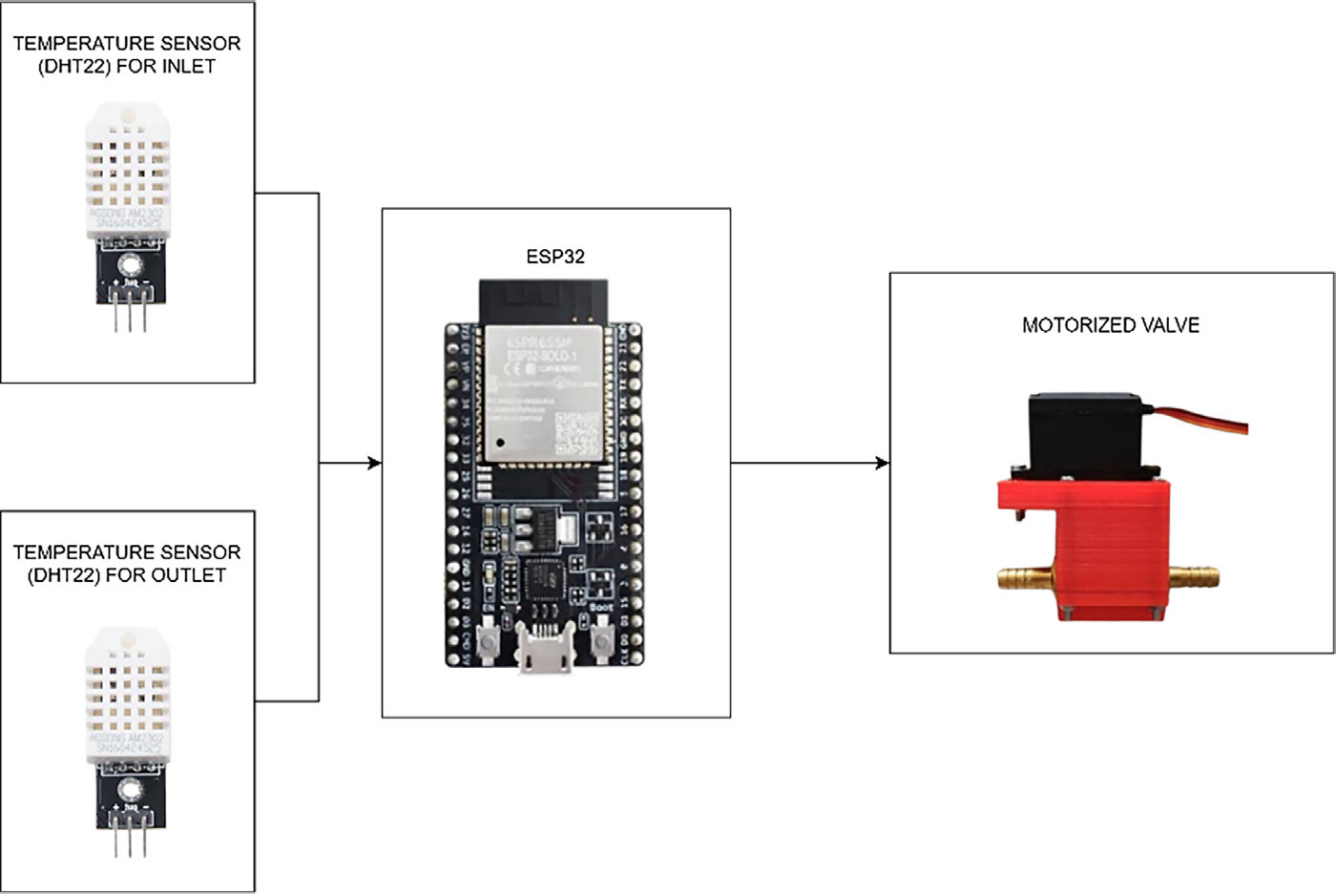
Schematic of control wiring.

Drying coffee beans using fuzzy logic control is supported by a series of controllers. The controller circuit is shown in Fig. 4. and is developed to run all the microcontroller components.

### Drying kinetics and mechanism

Drying is a complex process involving constant transfer of heat and mass. Drying occurs by evaporating water using heat and usually heat is flowed through a stream of hot air. Convection heat transfer is carried out to heat the outer surface of solids while conductive heat transfer allows heat penetration into the material [60], [61], [62]. After the evaporation of water from the solid, the mass of a material decreases, Because the physical properties of solids can change during drying, predicting the drying rate based on theoretical principles is often difficult [63], [64], [65]. Rate of drying defines as the rate at which the mass of water associated with a wet solid reduces with time (2):

(2) $$N=-M_{S}\frac{dX}{dt}$$

The rate of drying can also be expressed on a unit area basis as a flux $n_{a}$ on the Eq. (3):

(3) $$n_{a}=\;-\frac{M_{S}}{A}\frac{dX}{dt}$$

Where A is the area available for evaporation. Alternatively, the specific drying rate per unit mass of dry solid, $n_{m}$ on the Eq. (4):

(4) $$n_{m}=-\frac{dX}{dt}$$

With:

*N* = Rate of Drying [MT^−1^]
$n_{a}$ = Rate of Drying ($kg/m^{2}h$)
$n_{m}$ = Rate of Drying (kg/kgh)
$M_{S}$ = mass of completely dry solid (kg)
*X* = moisture content of the solid expressed on a dry mass basis
*t* = Time (h)

**Fig. 5:**
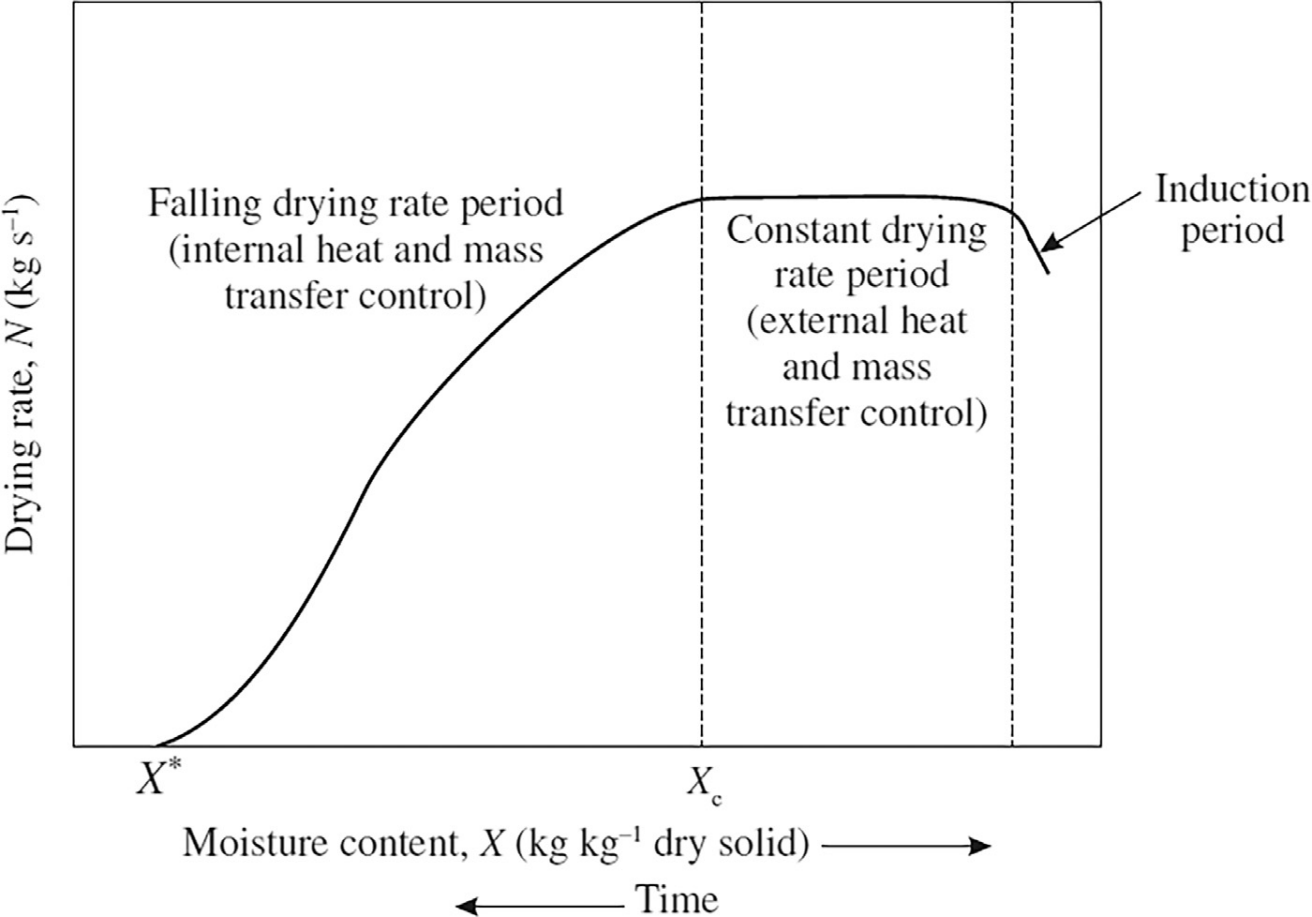
Drying rate curve for constant drying conditions [67].

Drying kinetics is evaluated by describing how quickly a material dries based on its moisture content (X) measured experimentally. The result is a drying rate curve, as seen in Fig. 5. The shape of this curve varies depending on the type of material, its size, thickness, and the applied drying conditions. The measurement of the drying rate curve is carried out by keeping the air temperature, humidity, flow rate, and flow direction constant [48,66]. This process only considers the gas phase and causes changes in moisture content as well as other properties of the dried solid [67].

## Fuzzy logic control

This section, the proposed FLC framework for the coffee bean dryer in a rotary dryer machine is discussed and presented.

### Mathematical model of fuzzy logic control

Fuzzy systems are nonlinear mappings between inputs and their outputs. The inputs and outputs of fuzzy are actual values, not fuzzy sets. The main components that make up a Fuzzy logic Controller are fuzzification units, which is a process to convert non-fuzzy variables (numerical variables) into fuzzy variables (linguistic variables), fuzzy inference is a process to get output from an input condition by following predefined rules, knowledge base and defuzzification units are processes to obtain numerical values from fuzzy data generated from the inference process. The interference method used in this study is the Mamdani method, often known as the min–max method. Defuzzification on the composition of Mamdani rules using the centroid method. The reason for using the Mamdani method is that the defuzzification value will move smoothly so that the change of a fuzzy set will also run smoothly.

**Fig. 6:**
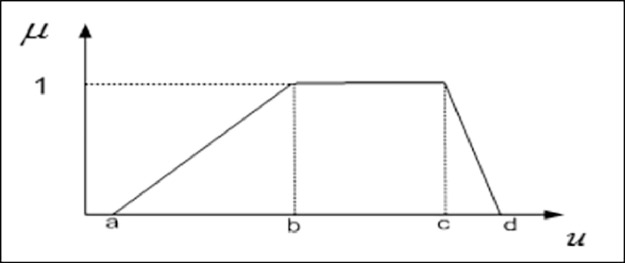
The trapezoidal fuzzy membership function.

The membership function notifies the truth value of the members of the fuzzy set. The interval of values used to define the membership function which is zero and one. Each membership function maps elements of crisp sets to the fuzzy set universe. The definition of a trapezoidal function is shown in Eq. (5) and the membership function of a trapezoidal shape is shown in Fig. 6.

(5) $$T\left(u;a,b,c,d\right)=\{\begin{array}{cc} 0 & u<a\\ \frac{u-a}{b-a} & a\leq u\leq b\\ 1 & b\leq u\leq c\\ \frac{d-u}{d-c} & c\leq u\leq d\\ 0 & d>u\; \end{array}\;$$

### Fuzzy logic design dan membership function

Fuzzy logic Control design as a heat flow controller on the coffee bean dryer with two inputs, error and delta error, and one output, valve opening. The error and delta error formulae are as follows:

(6) $$Error=\frac{T_{0}-T_{1}}{T_{0}}\times \;100\%$$

Dengan:

$T_{0}$ = Set point temperature (60 °C)
$T_{1}$ = Temperature read ( °C)

From formula (6) the delta error formula will be obtained as follows:

(7) $$\Delta Error=E_{1}-E_{0}$$

With:

$E_{1}$ = Read/actual errors (%)
$E_{1}$ = Previous errors (%)

**Fig. 7:**
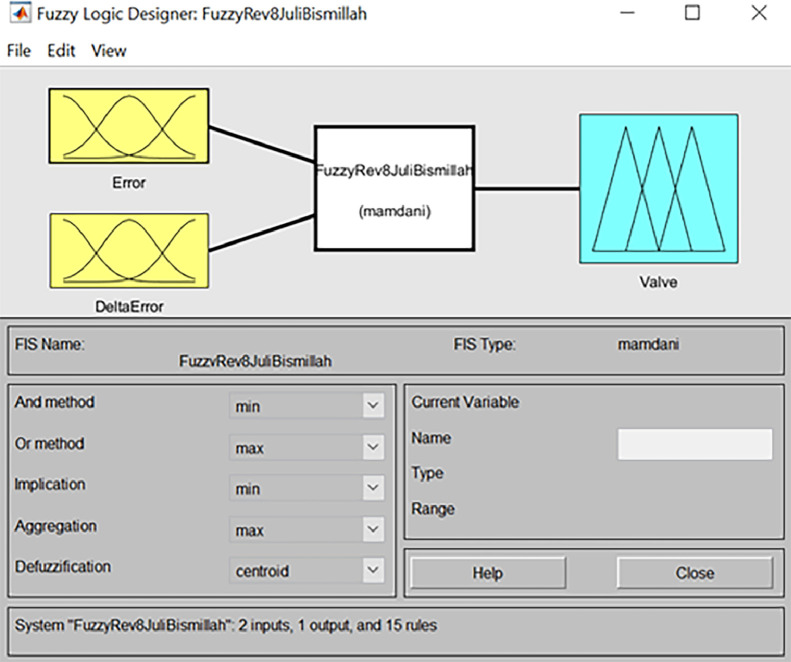
Fuzzy logic designer.

In Fig. 7, a fuzzy logic designer has been simulated using the matlab program.

The membership function is necessary in fuzzy logic to assess how much an input or output value possesses particular features or characteristics. In Fig. 7, there are two inputs, error and delta error, and the output is the valve opening.

**Fig. 8:**
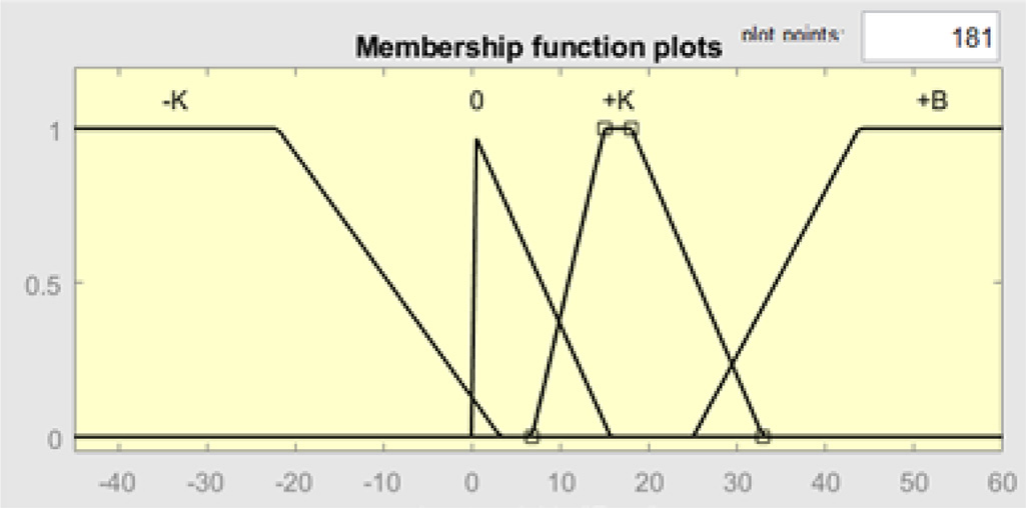
Membership function plot input error.

Fig. 8 above shows the error input membership function; there are four categories of membership functions, namely -K (Small in negative), 0, +*K* (Small in positive), and +*B* (Large in positive). Each of these categories has a specific range of values that represent the magnitude of the error. A negative error means that the temperature read is greater than the temperature of the set point, while a positive error means that the temperature read is below the set point. The value of 0 is the lowest error, which means the temperature in the desired setpoint. For the -K function has a range of values [−45 −45 −22 3.206], 0 [−0.0347 0 0 15.68], +*K* [6.771 15 18 32.9], and +*B* [25 43.75 60.65 60.65].

**Fig. 9:**
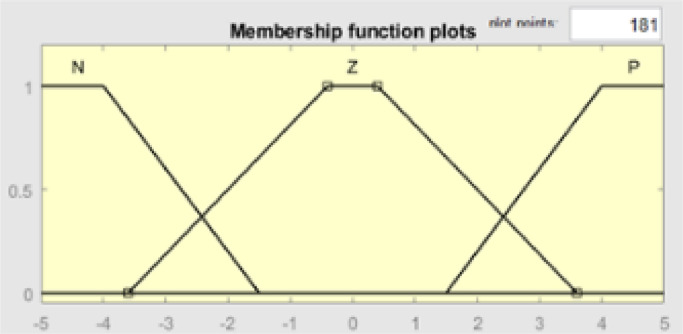
Membership function plot input delta error.

**Fig. 10:**
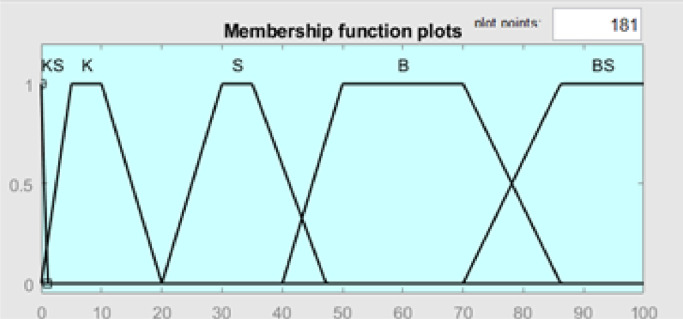
Membership function plot output valve.

The same applies to the input error delta shown in Fig. 9. The above 12 have three categories of membership functions, namely N(Negative), Z(Zero), and P(Positive). Each range of values in this category has a specific membership value that indicates the delta error of the system. The functions of the N member range from −5 to −1.5, the Z member from −3.6 to 3.6, and the P member from 1.5 to 5.5 Fig. 10.

The output of the membership function is the percentage of valves; there are five categories of membership functions, namely KS (Very Small), K (Small), S (Medium), B (Large), and BS (Very Large), which have a specific range of values that indicate the magnitude of the percentage of valves that are suitable for specific conditions. The membership value in the KS category is in the value range [−2 0 0 1], category K in the value range [0 5 10 20], category S at [20 30 35 47.28], category B at [40 50 70 86.18], and category BS at [70.2 86.4 100 100]. In this case, the membership function is essential in converting input values into output values using fuzzy inference methods. The ruled-based system used in this study contained 12 rules, namely (Table 1).

1. If error -K (Small Negative) and delta error N (Negative) then Valve KS (Very Small)
2. If error -K (Small Negative) and delta error Z (Zero) then Valve KS (Very Small)
3. If error -K (Small Negative) and delta error P (Positive) then Valve KS (Very Small)
4. If error −0 (Zero) and delta error P (Positive) then Valve B (Large)
5. If error −0 (Zero) and delta error N (Negative) then Valve S (Medium)
6. If error −0 (Zero) and delta error Z (Zero) then Valve S (Medium)
7. If error +*K* (Small Positive) and delta error N (Negative) then Valve B (Large)
8. If error +*K* (Small Positive) and delta error Z (Zero), then Valve B (Large)
9. If error +*K* (Small Positive) and delta error P (Positive), then Valve BS (Very Large)
10. If error +*B* (Large Positive) and delta error P (Positive), then Valve BS (Very Large)
11. If error +*B* (Large Positive) and delta error Z (Zero), then Valve BS (Very Large)
12. If error +*B* (Large Positive) and delta error N (Negative), then Valve B (Large)

**Table 1:** Rule Based Fuzzy Logic.

	Delta Error			
Error		N	Z	P
	-K	KS	KS	KS
	0	S	S	B
	+*K*	B	B	BS
	+*B*	B	BS	BS

### Ruler viewer fuzzy logic control

**Fig. 11:**
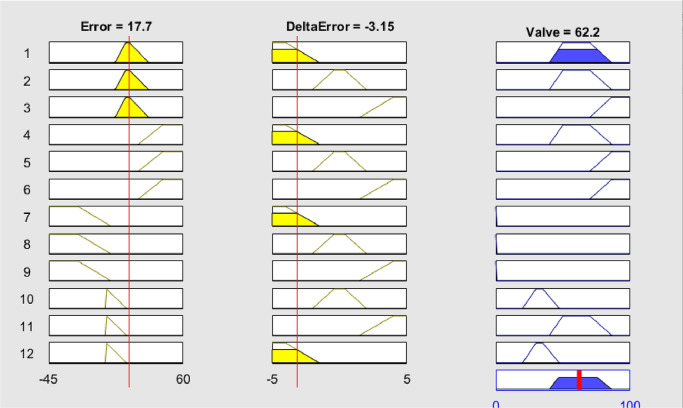
Rule-based viewer.

**Fig. 12:**
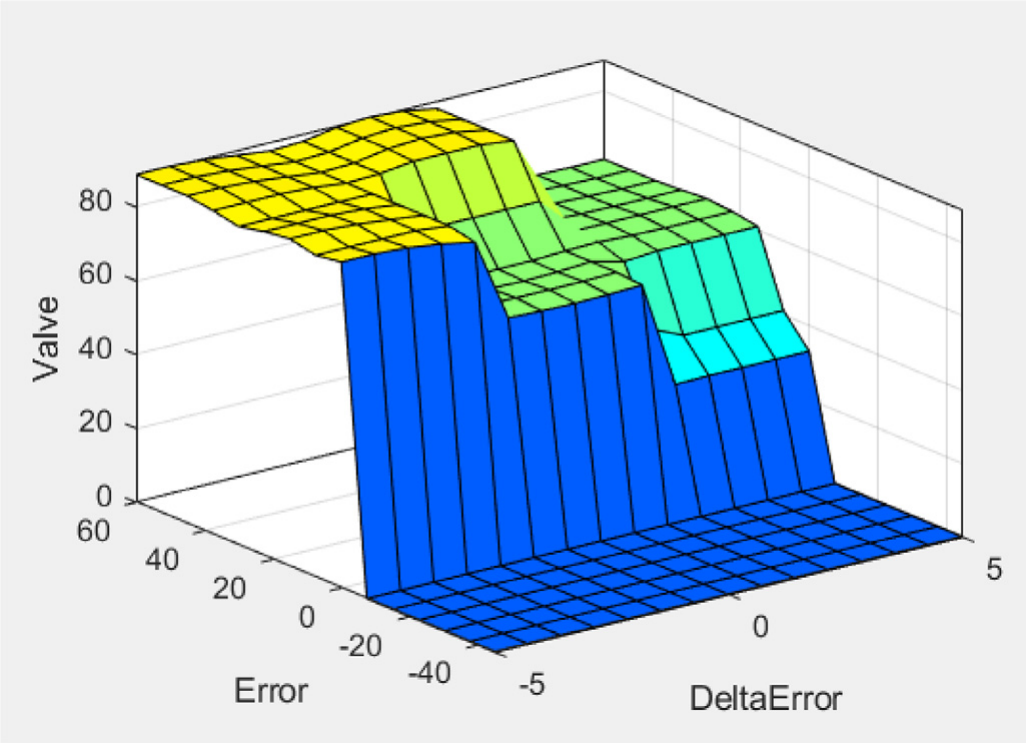
Surface viewer.

The following are the simulation results using Matlab using several examples of error parameters and error deltas Fig. 11, Fig. 12.

Overall, the surfaces displayed through the surface viewer show terraced surfaces such as stairs. There are different levels of surface elevation and color at specific elevation levels. The survey viewer is divided into four different color levels. A positive error will produce a high value marked with a yellow counture, a zero error will maintain the result in the middle with a light blue counture, and when a negative error will produce a meager value with a dark blue counter. A positive delta error means the temperature drops or is smaller than before, while a negative delta error means the temperature rises or is higher.

## Result and discussion

### Analysis of temperature test results - time

**Fig. 13:**
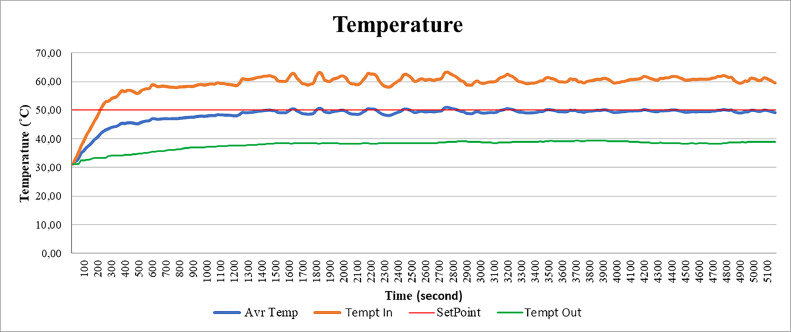
Graph of temperature against time.

In this section will explain the analysis of the graph between time and temperature. The temperature in this graph is divided into 3, namely the inlet temperature or the temperature when air enters the rotary dryer, the outlet temperature which is the temperature of the output side of the rotary and the average temperature of the two. In this case, the temperature that becomes the setpoint value is the average temperature of 50°C with a red line on the graph Fig. 13.

From Fig. 4, 8 values of inlet temperature when the average temperature has reached a temperature of 50 °C of 58 °C - 62 °C while the inlet temperature reaches 38 °C which means the difference between the inlet and outlet temperature is about 20 °C. The reason for using the average temperature is because the average temperature has the same value as the temperature in the middle of the rotary dryer which is where the coffee beans are dried.

### Analysis of valve temperature-percentage test results

**Fig. 14:**
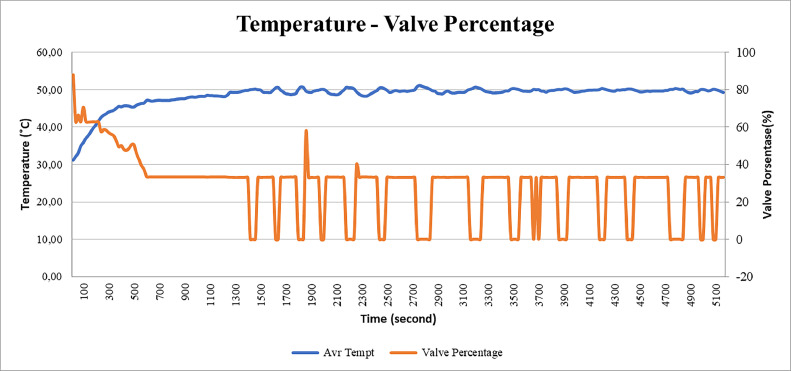
Temperature and valve graph with time.

In this section, we will explain the analysis of the graph between temperature and valve percentage. From Fig. 14 it can be seen that when the temperature is low, the error will be higher and positive, which means that when the error is high, the percentage of the valve will be very high as well. However, the percentage of valves will continue to decrease until it reaches 33%, where the point is the point when the error is 0% or the temperature has reached the set point.

At that point, the fire will be maintained in that state so that the percentage of valve will also be maintained at that value. However, if the temperature increases, the percentage of the valve will immediately drop to a value of 0% for the reason that the temperature can immediately drop quickly. Meanwhile, if the error has reached the 0% point again, then the valve percentage will rise again and be maintained at a value of 30%.

### Analysis of temperature-error test results

**Fig. 15:**
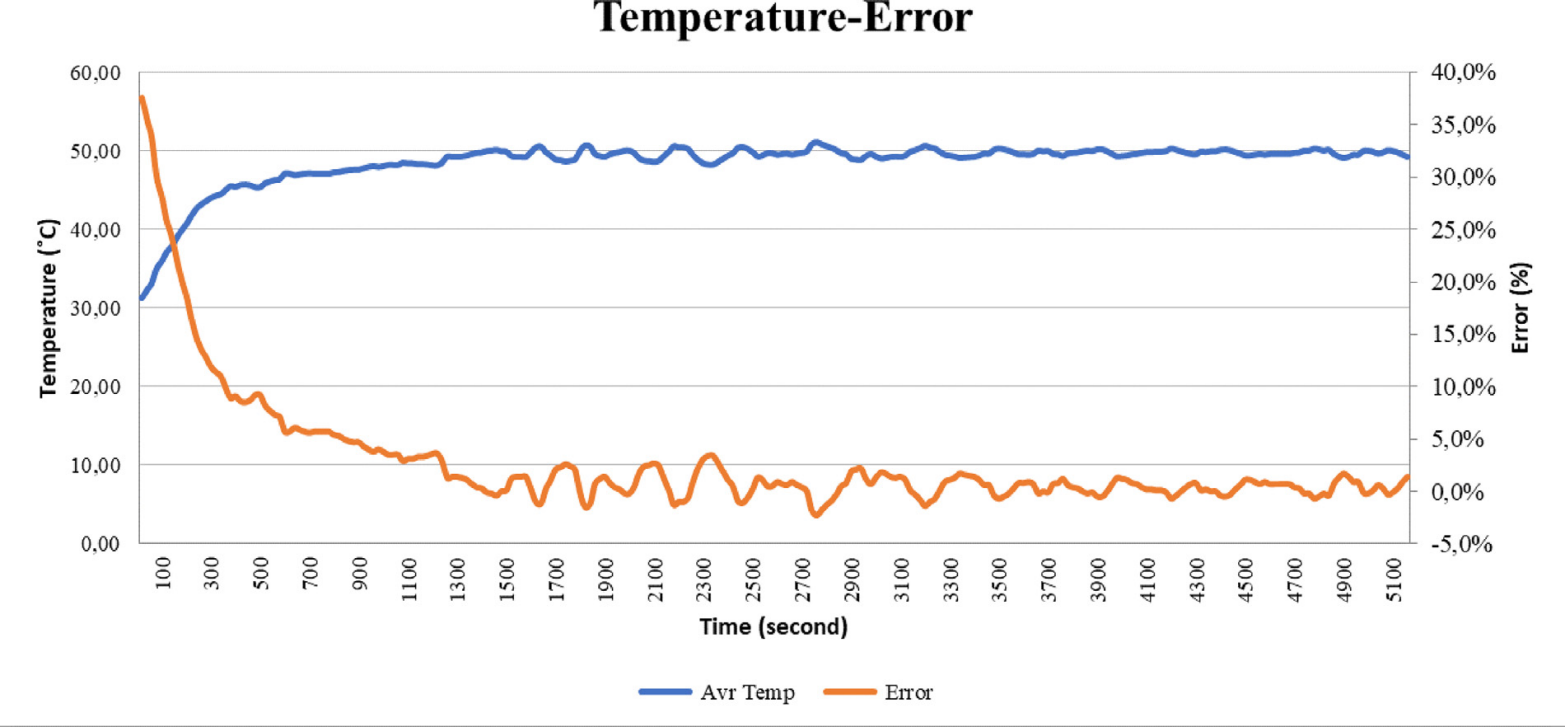
Graph temperature error and time.

In this section, we will explain the analysis of the graph between temperature and the error function. It can be seen from the graph in Fig. 15 that the temperature and error of the result will be inversely proportional. When the temperature is low or less than the set point, the error will be very high and when the temperature has reached the set point, the error will also decrease. An error with a positive value means that the temperature is still below the set point while an error with a negative value means that the temperature has passed the specified set point. Error is the main variable in determining the percentage of valves.

### Analysis of error-valve test results

**Fig. 16:**
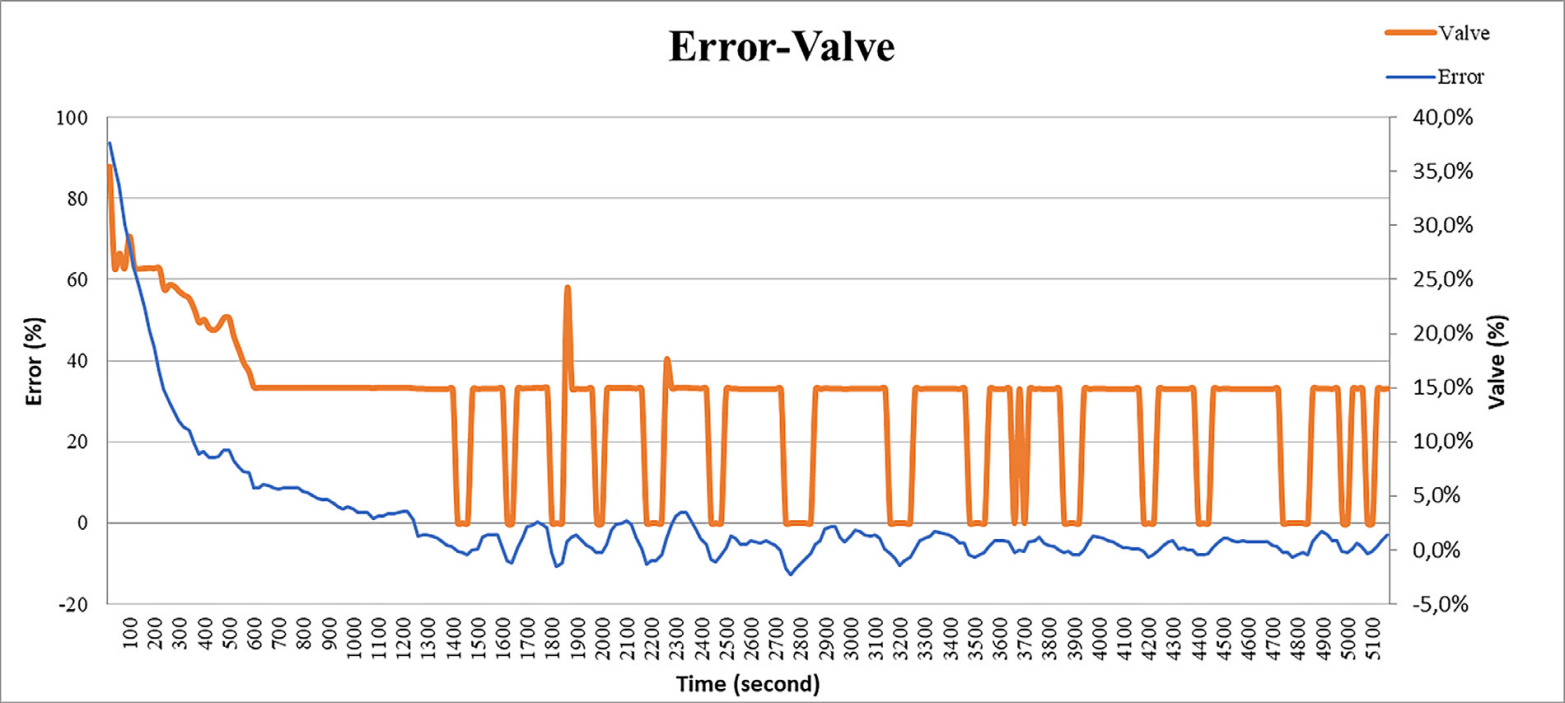
Error Graph and Valve with Time.

In this section, we will explain the analysis of the graph between temperature and the error function. The red line is an error value of 0% which means that the temperature has reached the set point. In the graph in Fig. 16 it can be seen that when the error is very high, the valve percentage will be very high as well. But when the error goes down, the percentage of valve will go down.

Errors in this case are divided into 2, namely positive errors and negative errors. The error is positive when the temperature has not reached the set point, so the valve percentage will increase, while the error is in a negative value, the valve percentage will decrease and the error is zero, meaning the valve will maintain its position at a low value so that the temperature is maintained according to the specified set point. It can also be seen that errors that have quite high spikes will also affect the percentage of valves with high spikes as well. This is caused by a high delta error.

### Analysis of temperature-error test results

**Fig. 17:**
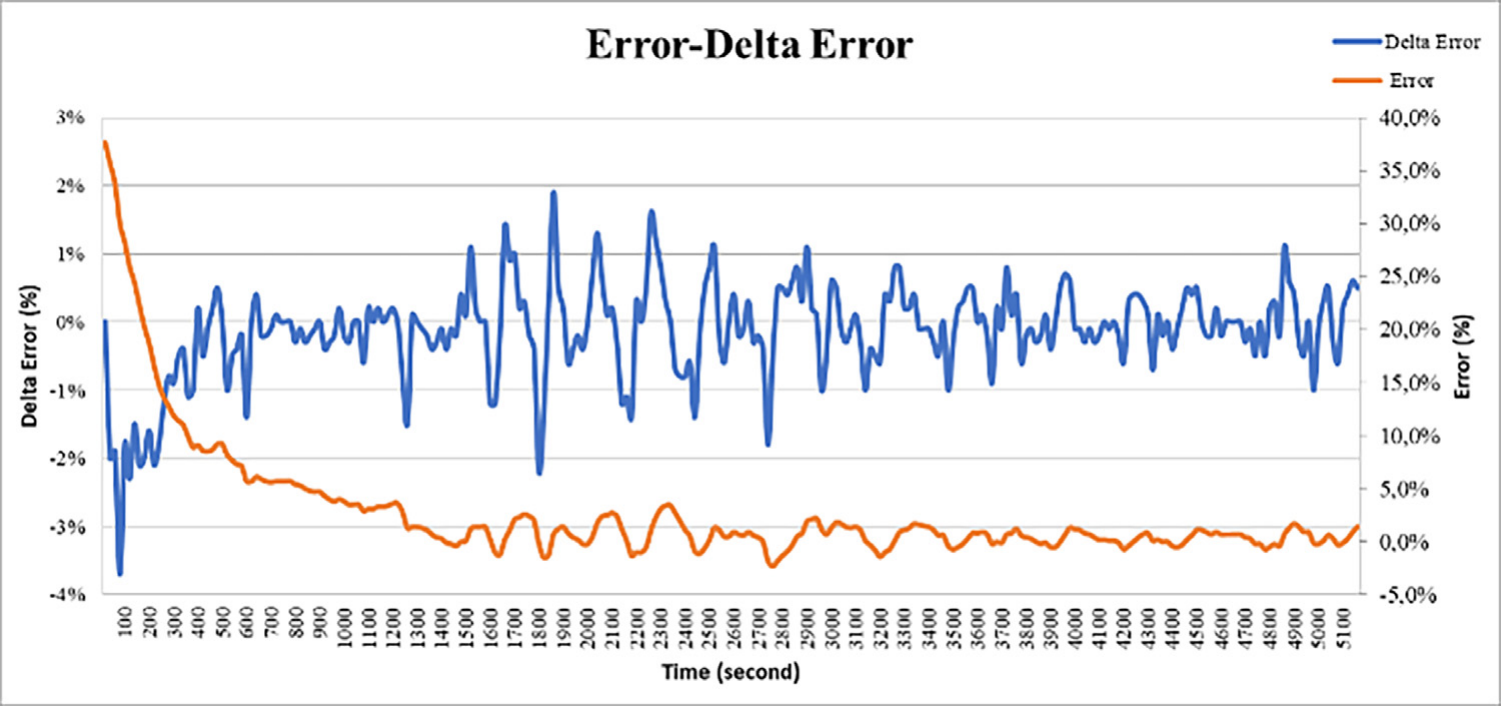
Error graph and error delta with time.

In this section, we will explain the analysis of the graph between errors and delta errors in Fig. 17 The blue line is the delta error line and the orange line is the error line, both of which are in percent form. Delta error is the result of a change in error, when the previous error of the error read has a considerable difference, then the delta error will be larger and vice versa if the error change is small then the error delta is also small. The delta error will also affect the percentage of valves produced.

### Quality characteristics of coffee test results

**Fig. 18:**
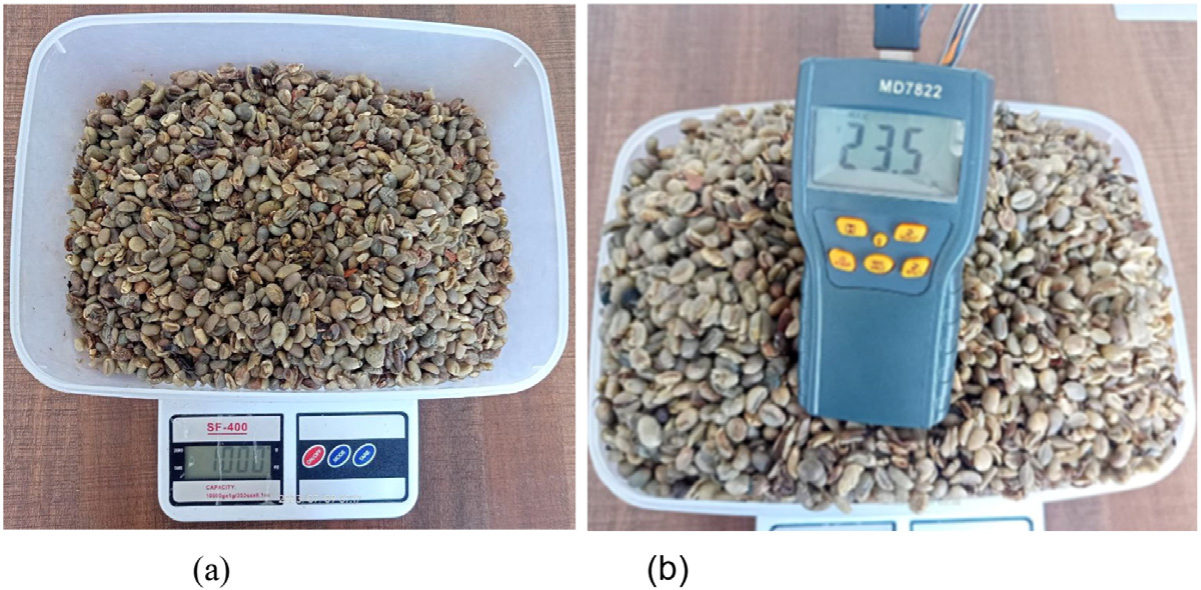
(a) Mass of coffee beans before drying (b) moisture content of coffee beans before drying.

**Fig. 19:**
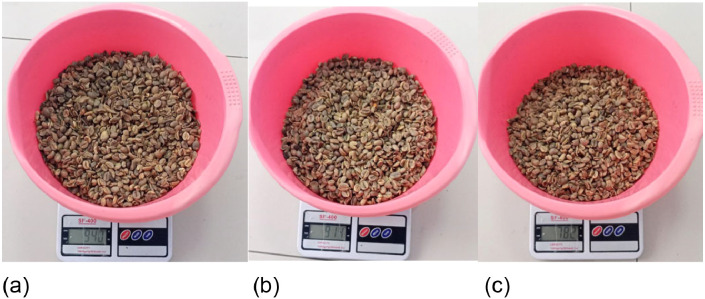
(a) Coffee bean mass drying 30 min (b) Coffee bean mass drying 60 min (c) Coffee bean mass drying 90 min.

Observation of the results of drying Robusta coffee beans based on temperature regulation treatment using error and delta error parameters, it can be observed that the moisture content of the material after drying varies for 30 min, 60 min and 90 min. The mass of coffee beans before drying of 1000-grams with a moisture content of 23.5% can be seen in Fig. 18, Fig. 19.

**Fig. 20:**
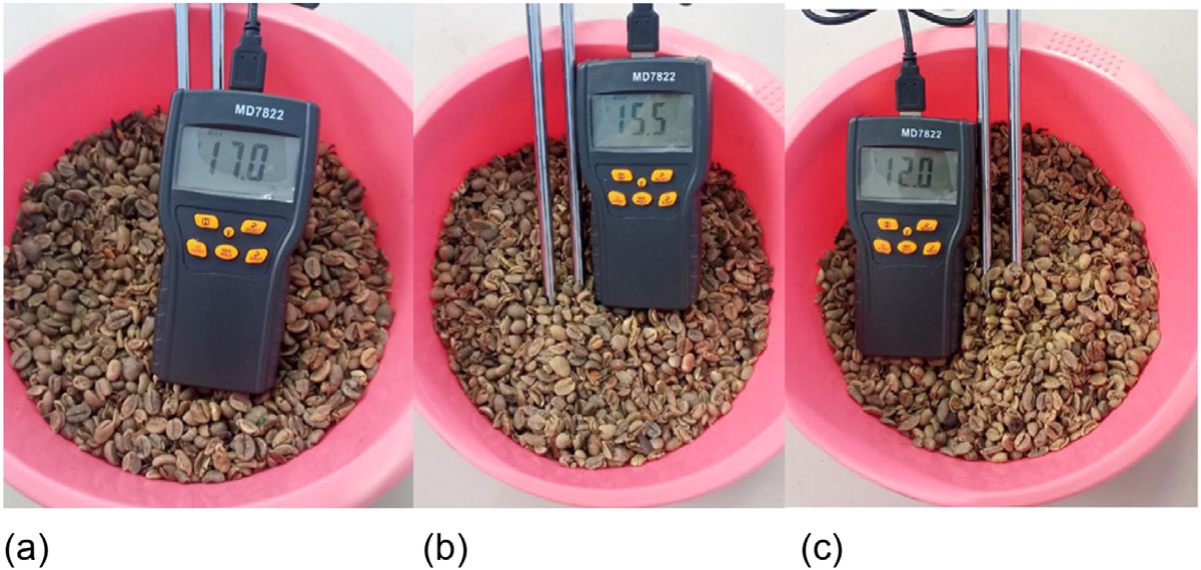
(a) Moisture content of coffee beans in 30 min drying (b) Moisture content of coffee beans in 60 min drying (c) Water content of coffee beans in 90 min drying.

The moisture content of dried coffee beans can be determined by analyzing the moisture content using a grain meter and calculating the decrease in moisture content using formula (8) The moisture content and mass of the product after drying are listed in Table 2 and in Figs. 19 and 20.

(8) $$Moisture\;Content\;Reduction=\frac{\left(m_{1}-m_{2}\right)}{\left(m_{1}-m_{0}\right)}\times \;100\%$$

**Table 2:** Experimental data.

Time (minute)	Initial Moisture Content (%)	Initial Mass (grams)	Final Water Content (%)	Final Mass (grams)	Moisture Reduction Calculation (%)
30	23,5%	1000	17%	940	6%
60	23,5%	1000	15,5%	914	9%
90	23,5%	1000	12%	784	22%

With:

$m_{0}$ = Weight of saucer and lid (grams)
$m_{1}$ = cup weight, lid and coffee snippet before drying (grams)
$m_{2}$ = Weight of cup, lid and coffee snippet after drying (grams)

Data collection at a certain period of time aims to determine the appropriate time to dry coffee beans in order to reach a minimum moisture content of 12.5% so that they meet SNI standards.

## Conclusion

Based on the research conducted by the author, the following conclusions can be drawn:

1. The resulting temperature control system uses fuzzy logic control interference Mamdani to control the temperature in the coffee bean dryer using a rotary dryer with a set point of 50°C. The algorithm used is MISO (Multiple Input Single Output) with 2 inputs, namely error and delta error and one output, namely the percentage of valve as an actuator to control temperature. Membership function error as many as 4 are -K [−45 −45 −22 3.206], 0 [−0.0347 0 0 15.68], +*K* [6.771 15 18 32.9], and +*B* [25 43.75 60.65 60.65] and membership function delta error as many as 3 namely N [−5.5 −5.5 −4 −1.5], Z [−3.6 −0.5 0.5 3.6] and P [1.5 4 5.5 5.5]. While the membership function percentage valve is 5, namely KS [−2 0 0 1], K [0 5 10 20], S [20 30 35 47.28], B [40 50 70 86.18], BS [70.2 86.4 100 100]. In addition, the rules used are as many as 12 rule based.
2. The test results used fuzzy logic control as a temperature controller on the dryer with an initial coffee moisture content of 23.5% and the semi-wash post-harvest method in the second drying within 30 min produced a moisture content of 17%, within 60 min produced a moisture content of 15.5% and within 90 min produced a moisture content of 12%. So it can be concluded that for a mass of 1kg with the post-harvest method of coffee is a semi wash can achieve drying according to the Indonesian National Standard (SNI 01–2907–2008) is within 90 min. While the results of water content with the on-off method within 90 min produce a final moisture content of 12.5%. This value is still within the standard threshold of expected moisture content. However, the fuzzy logic control method has advantages in setting the temperature according to the set point of 50°C compared to the on-off method which produces fluctuating temperatures between 45°C-50°C.
3. The estimation of drying time with varying masses was calculated using a system of linear equations by comparing the drying rate values between the masses of 1kg and 5kg in the on-off control experiment. The drying rate value at a mass of 1kg is 0.0025%/s and at a mass of 5kg is 0.00235/s so that a decrease in drying rate of 0.00015%/s is obtained for each mass of 1kg. From this value, the time to dry coffee beans as much as 10kg is 195.65 min.

## Declaration of competing interest

The authors declare that they have no known competing financial interests or personal relationships that could have appeared to influence the work reported in this paper.

## Data Availability

Data will be made available on request.
